# Endophytic fungus *Phomopsis liquidambari* and different doses of N-fertilizer alter microbial community structure and function in rhizosphere of rice

**DOI:** 10.1038/srep32270

**Published:** 2016-09-06

**Authors:** Md. Ashaduzzaman Siddikee, Mst Israt Zereen, Cai-Feng Li, Chuan-Chao Dai

**Affiliations:** 1 Jiangsu Key Laboratory for Microbes and Functional Genomics, Jiangsu Engineering and Technology Research Center for Industrialization of Microbial Resources, College of Life Sciences, Nanjing Normal University, Jiangsu Province, China

## Abstract

Microbial community structure and functions of rhizosphere soil of rice were investigated after applying low and high doses of nitrogenous fertilizer and *Phomopsis liquidambari*. Average well color development, substrate richness, catabolic diversity and soil enzymes activities varied after applying N-fertilizer and *P. liquidambari* and were greater in *P. liquidambari* treated soil than only N-fertilization. Multivariate analysis distinctly separated the catabolic and enzymes activity profile which statistically proved alteration of microbial functional diversity. Nitrogen fertilizer altered microbial community structure revealed by the increased content of total PLFAs, specific subgroup marker PLFAs except fungal PLFAs and by the decreased ratio of G^+^/G^−^, sat/monunsat, iso/anteiso, F/B except trans/cis while *P. liquidambari* inoculation enhanced N-fertilization effect except increased fungal PLFA and decreased trans/cis. PCA using identified marker PLFAs revealed definite discrimination among the treatments which further statistically confirmed structural changed of microbial community. Nitrogenase activity representative of N-fixing community decreased in N-fertilizer treatment while *P. liquidambari* inoculation increased. In short, application of *P. liquidambari* with low doses of N-fertilizer improved rice growth and reduced N-fertilizer requirement by increasing enzymes activities involved in C, N and P cycling, structural and functional diversity of microbes, nitrogenase activity involved in N_2_ fixation and accumulation of total-N.

Rice (*Oryza sativa* L.) is the second largest crop in terms of planted area and yield in the world. The cultivation of rice gives rise to anoxic conditions in growing season but aerobic conditions during non-growing season^1^. Thus, composition and structure of microbial community in rice field are diverse and very complicated^2^. Agricultural management practices, particularly inputs of fertilizers and manure, tillage, cover crops, cropping system and season, soil pH and application of plant growth promoting bacteria or fungi have large impacts on the size and activity of soil microbial communities^3^,^4^,^5^,^6^,^7^,^8^. Nitrogen is the top most important macronutrients and limiting factor for plant growth and development^9^. Soils are routinely fertilized to overcome N-limitation and maximize crop yield. But, repeated fertilizer applications change soil physical, chemical and biological properties consequently directly or indirectly influence the growth and activity of certain group of microbes and adaptation ability of different groups of microbes varies with nutritional status^10^,^11^. The rhizosphere of plant itself is also a unique ecological niche and shapes the structure of microbial community by releasing specific substrates or by the specific physical, chemical and biotic environment created by the plant root in terms of O_2_, pH etc.^12^,^13^. Quality and quantity of root exudates depends on plant species, nutrient levels, biotic and abiotic stresses and colonization by PGP microorganism^3^,^14^. *P. liquidambari* colonization increased the concentration of soluble saccharides, total free amino acids and organic acids in rice roots exudates and shape community structure^3^. Hecke *et al*.^15^ too reported *N. coenophialum* enhanced rhizodeposition by tall fescue, consequently influence microbial population^15^. Our previous study reported that *P. liquidambari* affects nitrogen transformation processes in rice rhizosphere by shifting microbial community involved in N-turnover^3^,^16^,^17^ and by affecting microbial abundance of AOA, AOB and diazotrophs^3^. But those studies emphasized only on microbial community related with N-processing such as AOA, AOB and *nif*H and were unable to clarify the extent of changes at the whole community level in respect of function and structure. We therefore attempted to get the clear picture about the effect of application of N-fertilizer with or without *P. liquidambari* on whole community structure, functional diversity, extra-cellular enzymes activity, N-fixation efficiency and growth of rice plant.

Changes in soil microbial functional diversity has been frequently analyzed by community level physiological profiling^18^ and extracellular enzyme activity^4^,^19^. Biolog EcoPlate technique proposes a simple and sensitive way for CLPP to compare potential metabolic diversity^20^ among various vegetations^21^,^22^. The shifts in BIOLOG metabolic diversity represent the shifts in community composition^23^. Therefore, we used EcoPlate to compare potential metabolic shift and diversity of microbial communities of rhizospheric soil of rice after applying different doses of N-fertilizer with or without *P. liquidambari*. Soil enzymes activities involved in C-, N-, P-, and S-cycling represent functional diversity of microbial communities^19^. Mineral fertilizers have positive, negative or neutral effects on activities of soil C-, N-, and P-cycling enzyme^24^ but AMF and PGPR enhanced enzyme activities such as urease, dehydrogenase and phosphatase^25^. Our previous study demonstrated that *P. liquidambari* accelerated leaf litter decomposition and N-transformation^26^ but still require more understanding regarding the effect of *P. liquidambari* on soil enzymes involved in C, N and P cycling. As soil enzyme activities have potential to provide a unique integrative assessment of soils functional changes so the influences of *P. liquidambari* and N-fertilization on soil enzyme activities were investigated.

Phospholipid fatty acid primarily are polar lipids; essential component of cell membrane of microbes and widely used for evaluating microbial community structure. These compounds rapidly decompose as soon as cells die, therefore, PLFA are good indicators of living organisms^27^. Moreover, the chemical composition of PLFAs differs in different organisms and signature PLFAs can provide information on specific groups of microbes^28^. The changes in specific marker PLFAs in PLFA profiles described the changes of specific group as well as overall structure of microbial communities^29^. Furthermore, changes the ratios of sat/monouns, fungi/bacteria, G^+^/G^−^, iso/anteiso and trans/cis PLFAs can be used to described changes of community structure^29^,^30^. Therefore, we used PLFA technique to study the changes in specific PLFA markers representative of specific microbial groups to describe influence of different doses of N-fertilizer and *P. liquidambari* on community structure of rhizosphere soil of rice.

*P. liquidambari* produce indoleacetic acid and abscisic acid *in vitro*^31^,^32^, forms symbiotic relationship with rice plant, stimulates the expression of several genes involved in N-uptake and metabolism^16^ consequently enhanced N accumulation and N use efficiency of rice^17^, significantly reduces the requirement of N-fertilizer and promotes growth, increase biomass and yield^16^,^17^. Yang *et al*.^3^ reported better growth of rice after applying low level of N-fertilizer and *P. liquidambari* but abundance of AOA, AOB, and N_2_ fixing diazotrophs were higher in the rhizosphere soil treated with high doses of N-fertilizer^3^. Nitrogenase enzyme responsible for N_2_ fixation also reduced C_2_H_2_ (acetylene) to C_2_H_4_ (ethylene)^33^ provides a highly sensitive and inexpensive way to quantify N_2_ fixation and nitrogenase activity. Thus, we performed acetylene reduction activity assay to discriminate efficiency of N-fixing community belong to low and high doses of N-fertilizer with or without *P. liquidambari* inoculation. Therefore, the aim of this study was to determine the effects of different doses of N-fertilizer with or without *P. liquidambari* inoculation on (i) microbial community structure, function and extracellular enzyme activities, (ii) N_2_ fixation, nitrogenase enzyme activity and N-fixing community and (iii) growth promotion of rice.

## Results and Discussions

The physical and chemical properties of soil changes very slowly due to environmental fluctuations whereas biological properties react quickly and they are sensitive even to small fluctuations. Therefore, we used the changes of microbial community structure and function of soil as potential indicator of ecosystem health of rice field after applying different doses of N-fertilizer and *P. liquidambari* for short term experiment.

### Effect of different doses of N-fertilizer and plant growth promoting fungus *P. liquidambari* on community level physiological profiles (CLPP)

**Figure 1:**
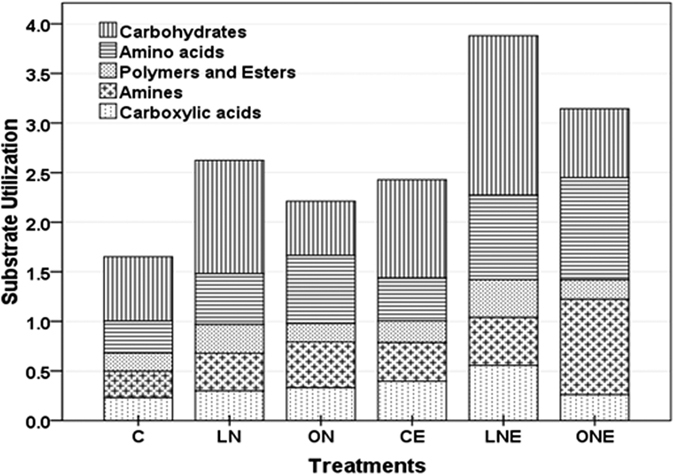
Abundance of substrate utilization by soil microbial community of rice rhizosphere cultivated under different level of N-fertilizer with and/or without inoculation of endophytic fungus *Phomopsis liquidambari* as evaluated in the Biolog EcoPlate incubated for 96 h. Rice seedling subjected under the following six treatment vs C: Control; LN: Low doses of N-fertilizer; ON: High doses of N-fertilizer; CE: Control + *P. liquidambari*; LNE: Low doses of N-fertilizer + *P. liquidambari*; ONE: High doses of N-fertilizer + *P. liquidambari*.

**Figure 2:**
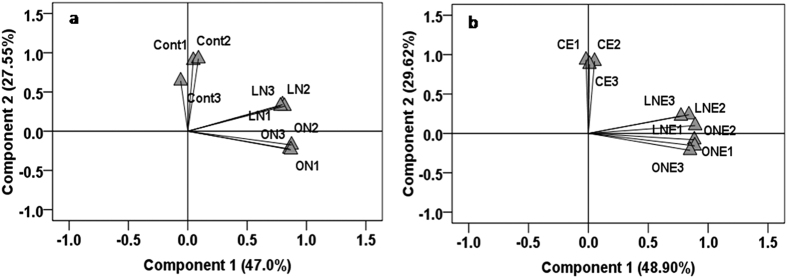
Discrimination of microbial community by principal component analyses (PCA) based on carbon substrate utilization patterns of Biolog Ecoplate data incubated for 96 h of rice rhizosphere treated with different level of N-fertilizer with and/or without inoculation of endophytic fungus *Phomopsis liquidambari*. Rice seedling subjected under the following six treatment vs C: Control; LN: Low doses of N-fertilizer; ON: High doses of N-fertilizer; CE: Control + *P. liquidambari*; LNE: Low doses of N-fertilizer + *P. liquidambari*; ONE: High doses of N-fertilizer + *P. liquidambari*.

Average well color development (AWCD) generally followed sigmoidal pattern with incubation time, but the rate of increase varied with different level of N-fertilizer application and *P. liquidambari* inoculation which indicating microbial composition and metabolic activity differ among treatment (Supplementary Table S1; Supplementary Fig. S1a,b). Garland^34^ suggested that the rate of color development in wells related with the density as well as metabolic activity of microbes in inoculums^34^. After 96 h, AWCD decreased sharply in ON and ONE treatment which could be due to the microbial community dominated by fast-growing microbes and elimination of some slow-growing microbes but other treatments did not show decreasing trend until 120 h. Slow-growing microorganisms such as fungi and actinomycetes are usually out-competed by the fast-growing bacteria in the Biolog assay^35^. In general, the microbial community belongs to rhizosphere soil of rice, treated with *P. liquidambari* and different doses of N-fertilizer had the higher AWCD values compared to non-inoculation as well as control (Supplementary Fig. S1; Supplementary Table S1). AWCD was higher in LNE followed by ONE and lowest in control (Supplementary Table S1; Supplementary Fig. S1a,b), suggesting that low doses of N-fertilizer and *P. liquidambari* inoculation favor total microbial abundance and activity. Our assumptions are in agreement with the report that color development depends on both the number and biomass of cells and their activity reflect physiological state of those cells^34^. Utilization of carbohydrate, carboxylic acids and polymer-esters was highest in *P. liquidambari* and low doses of N-fertilizer treated soil whereas lowest in soil treated with high doses of N-fertilizer irrespective of *P. liquidambari* inoculation (Fig. 1). Amino acids and amines utilization were highest in soil treated with high doses of N-fertilizer irrespective of *P. liquidambari* inoculation compared to other treatments (Fig. 1). The number of substrates used reflects the diversity of carbon-oxidation pathways and functional diversity^23^. Out of 31 kinds of sole carbon substrates utilization, highest number (25) was observed in the microbial communities belong to LNE soil and the lowest (18) number was observed in ONE and ON (Supplementary Table S1). Combined application of N-fertilizer and *P. liquidambari* increase the substrate use efficiency of microbial community but N-fertilizer alone had changed the carbon utilization profiles by decreasing the carbon metabolic activity, metabolic richness, and metabolic diversity of soil microbial community (Supplementary Table S1). Highest substrate utilization (Fig. 1), substrates richness (S = 25.56) and functional diversity (H = 3.22) were detected in low doses of N-fertilizer and *P. liquidambari* inoculated treatment (Supplementary Table S1) which might be due to the rhizosphere environment facilitated the growth of all microbes at the same extent belongs to communities with stronger metabolic activity to utilize the different carbon substrates. *P. liquidambari* and *N. coenophialum* symbiosis with rice and tall fescue, respectively enhanced rhizodeposition and consequently influence microbial population^3^,^15^. Substrate utilization (Fig. 1) except amino acid and amines, substrate richness (S = 18.11) and functional diversity (H = 2.90) of microbial community were lowest in ON and ONE (Supplementary Table S1; Fig. 1) which might be due to reduced diversity of microbes in the rhizosphere of rice by facilitating available N for existing community consequently become dependent on supplied N and losses ability for competitiveness. Fertilization certainly influences growth and activity of certain group of microbes^10^,^11^,^19^. Adaptation ability of different groups of microbes varied with the nutritional status and stress reduced biodiversity through extinction of sensitive species. The largest 13 carbon substrate utilization differences of microbes were in soils treated with high doses of N-fertilizer (Supplementary Table S1). Wang *et al*.^24^ recommended that lower carbon metabolism function of soil microbial community is a signal of degradation of soil ecosystem^24^. Further, separate PCAs were performed for the catabolic profiles of two set treatments; control, low and high doses of N-fertilizer (Fig. 2a) or *P. liquidambari* inoculation in control, low and high doses of N-fertilizer (Fig. 2b), which were clearly dissimilar and separated into three distinct groups; Fig. 2a, showing that the PC1 with 47% variance separated carbon substrate utilizing profiles of N-fertilizer and control treatments and PC2 with 27.55% variance separated different doses of N-fertilizer; In Fig. 2b, PC1 explained 48.90% variance between *P. liquidambari* inoculated N-fertilizer treatment and non-fertilized, however, PC2 explained 29.62% (about 2% higher) variance between CLPP of different doses of N-fertilizer inoculated with *P. liquidambari* which implied that the functional diversities of soil microbial community altered due to N-fertilizer application and *P. liquidambari* inoculation even separated at the level of their doses of application. The separation remain almost in the same quarter might be due to the short duration of the experiment. The catabolic profiles of the three replicates were almost merged together, suggested that the rhizosphere of rice of each treatment had relatively same microbial community, pattern of function and environmental condition (Fig. 2a,b). Numerous studies reported differences in carbon substrate utilization under various treatment including fertilizer amendments^19^,^21^,^22^,^23^.

### Effect of different doses of N-fertilizer and plant growth promoting fungus *P. liquidambari* on soil extra-cellular enzyme activities

**Figure 3:**
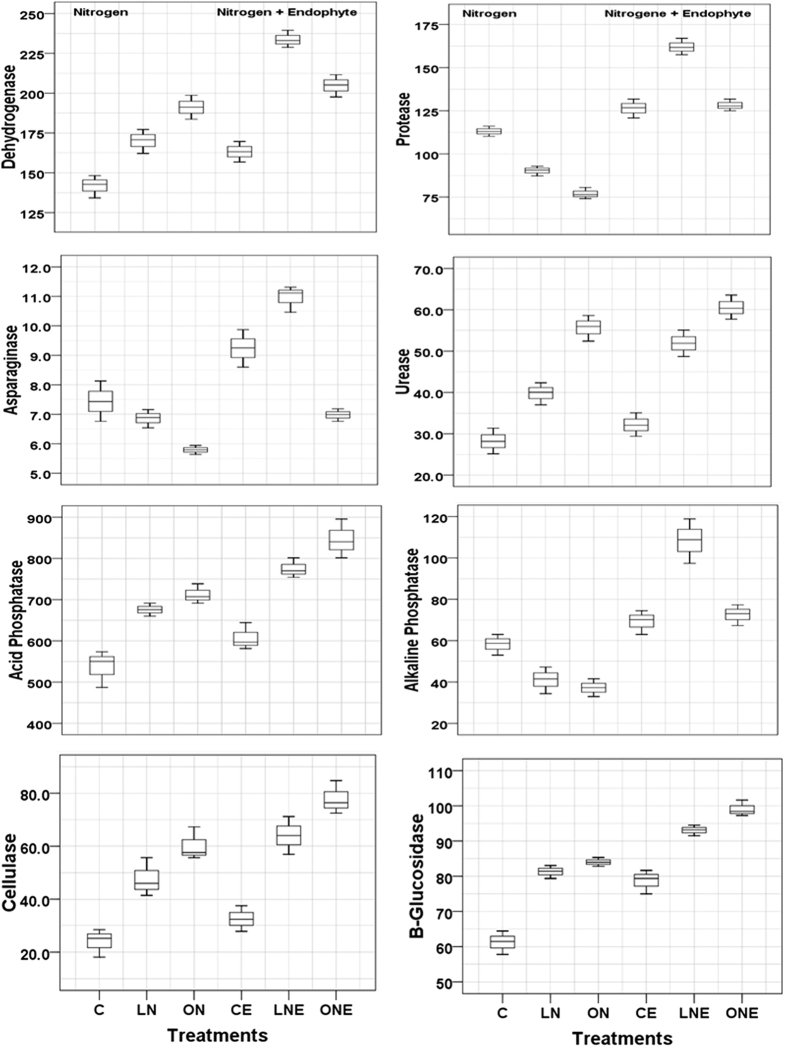
Box plots of potential enzyme activities of rhizosphere soil of rice treated with different doses of N-fertilizer with or without inoculation of *Phomopsis liquidambari*. Rice seedling subjected under the following six treatment vs C: Control; LN: Low doses of N-fertilizer; ON: High doses of N-fertilizer; CE: Control + *P. liquidambari*; LNE: Low doses of N-fertilizer + *P. liquidambari*; ONE: High doses of N-fertilizer + *P. liquidambari*. Dehydrogenase = mg TPF (kg air dry soil)^−1^ h^−1^; Protease = mg tyrosine (kg air dry soil)^−1^ h^−1^; Urease and asparaginase activity as mg NH_4_^+^-N g^−1^ soil h^−1^; β-glucosidase and phosphatase (acid and alkaline) = mg pNP (kg air dry soil)^−1^ h^−1^; Celllulase, amylase and invertase = mg glucose (kg air dry soil)^−1^ h^−1^. Note of Box plot: The horizontal line is the mean, and upper and lower “hinges” are the first and third quartiles, respectively. Upper and lower “whiskers” extend to the highest or lowest value, respectively.

**Figure 4:**
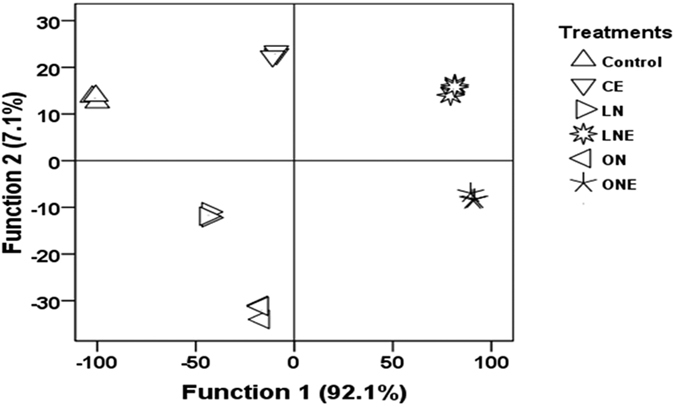
Discrimination function analyses (DFA) of the enzymes activity profiles of the rhizosphere soil samples of rice treated with different doses of N-fertilizer and *P. liquidambari*. Rice seedling subjected under the following six treatment vs C: Control; LN; Low doses of N-fertilizer; ON: High doses of N-fertilizer; CE: Control + *P. liquidambari*; LNE: Low doses of N-fertilizer + *P. liquidambari*; ONE: High doses of N-fertilizer + *P. liquidambari*. The different letter on the top of the column indicates a statistically significant difference between the treatments at P < 0.05 level using one-way ANOVA.

Soil enzymes are usually sensitive to disturbances in ecosystem^36^ and strictly correlated with its microbial population’s composition^37^. So, changes in enzymatic activity could be the indicator of the changes occurring in the microbial community under a wide range of management practices. The activities of 8 extracellular enzymes were quantified from rhizosphere soil of rice treated with two different levels of N-fertilizer and *P. liquidambari* (Fig. 3). In general, the activities of extracellular enzymes were greater in the rhizosphere soil of rice inoculated with *P. liquidambari* than the other soil irrespective of N-fertilizer application (Fig. 3). Low and high doses of N-fertilization in rice increased the activities of dehydrogenase related to microbial population size; cellulase and β-glucosidase involved in C-cycling; acid-phosphatase related to P-cycling and N-cycle urease whereas decrease the activities of protease, asparaginase and alkalaline phosphatase compared to control (Fig. 3). Increased dehydrogenase activities in submerged rhizosphere soil recorded after application of mineral fertilizer which indicates increased microbial activity. Mineral fertilizer increased dehydrogenase activity^38^ and an important indicator of microbial activity in submerged soils^39^. *P. liquidambari* inoculation in low and high doses of N-fertilizer treated soil further increased the dehydrogenase activities compared to uninoculation (Fig. 3). Enhanced dehydrogenase activity reported after application of AMF and PGPR^25^, compost and FYM compared to mineral fertilizer^40^. Similar to our result, cellulase and *β-*glucosidase activity increased in response to N-fertilization^41^,^42^ however further increase observed when *P. liquidambari* inoculated like application of AMF and PGPR^25^ (Fig. 3). Urease has been involved in urea hydrolysis and increases the utilization rate of N-fertilizer^4^. Urease activities increased with increasing doses of N-fertilizer and *P. liquidambari* inoculation further enhanced compared to corresponding control (Fig. 3). Increased urease activity reported after co-inoculation of *A. brasilense* and *M. oryzae*^43^ and AMF and PGPR^25^. Low and high doses of N-fertilizer application decrease the activities of protease, L-asparaginase and alkalaline phosphatase compared to control (Fig. 3) which might be due to the application of urea increase availability of N resulting reduced N demand from organic sources, fluctuation of soil chemical properties such as pH, reduced efficiency of these extra-cellular enzyme. Higher inorganic-N availability reduced activity of enzymes involved in N-mineralization^4^. Application of chemical fertilizers decreased soil pH in planted rice under flooded condition^44^ which might cause of reduction of alkalaline phosphatase activities. Acid phosphatase activity in different treatments was higher than that of alkaline phosphatase (Fig. 3) which might be due to the low soil pH as well as reduction of pH by N-fertilizer application^41^. *P. liquidambari* inoculation in rice treated with low doses of N-fertilizer increased the protease, asparaginase and alkalaline phosphatase activities (Fig. 3). Previously Chen *et al*.^26^ documented that *P. liquidambari* promotes organic matter decomposition and N-release via mineralization^26^,^45^. The score plots of discrimination function analyses showed that control treatment are distinct from CE located in 3^rd^ quarter, LN from ON in 4^th^ quarter and LNE in 2^nd^ quarter from ONE in 1^st^ quarter (Fig. 4). The DFA analysis clearly illustrated the differences in soil enzyme activities representing microbial community function belong to different doses of N-fertilizer and *P. liquidambari* inoculation treatments (Fig. 4). The application of lower doses of N-fertilizer and *P. liquidambari* for rice growth significantly increased most enzymes activities which might be due to the less harmful effect of low doses of N-fertilizer on extracellular enzyme and *P. liquidambari* inoculation increase the amount, type and composition of exudates in rhizosphere and allow a greater number of species to be competitive and higher functional diversity compared to other treatments. Root exudates stimulate the growth of microbes and activities of extracellular enzymes capable of influencing biogeochemical cycling of C, N, P and S^46^ and higher activity of several enzymes can be interpreted as a higher functional diversity of microbial community^19^,^47^.

### Effect of different doses of N-fertilizer and plant growth promoting fungus *P liquidambari* on microbial community structure

The PLFAs patterns varied significantly in response to different doses of N-fertilizer (C; 35.49, LN; 39.09 and ON; 44.23 nmol g^−1^ soil) and *P. liquidambari* inoculation (CE; 37.0, LNE; 50.79 and ONE, 47.32 nmol g^−1^ soil), as revealed by the total content of PLFAs (Supplementary Fig. S2). Fertilization increased total PLFA and was higher in fertilized land than unfertilized^29^. Total PLFA content were 30%, 12.90% and 6.82% higher in LNE compared to control, ON and ONE, respectively which might be due to the inoculation of *P. liquidambari* increase exudation by rice root and low doses of N-fertilizer optimize C/N ratio for microbial growth. *P. liquidambari* colonization altered the exudation of organic compounds by rice roots, effectively alters the balance of soil microbes in the rhizosphere^3^. Root exudates stimulated bacterial proliferation, microbial activity and communities^48^. The PLFA profiles were dominated by the group of saturated fatty acids that constituted 53.58–58.71% while unsaturated fatty acids contribute 41.28–46.41% of the total fatty acids detected in the soils (Supplementary Fig. S2).

**Figure 5:**
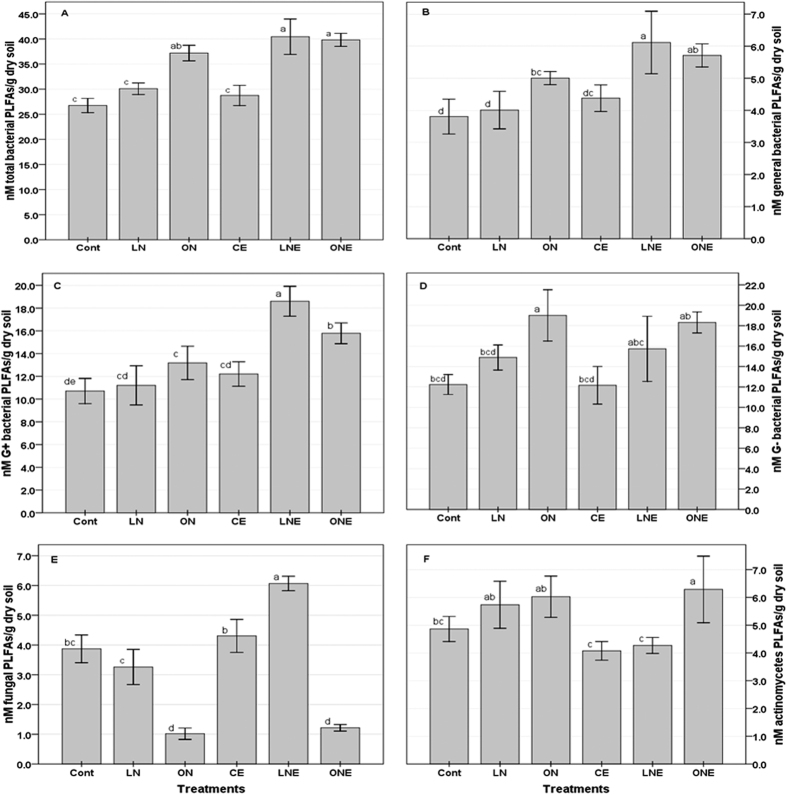
Abundance of total bacterial, general bacterial, gram-positive and gram-negative bacterial; actinomycetes and fungal PLFAs from rhizosphere soil of rice under different doses of N-fertilizer treatments with and/or without *P. liquidambari* inoculation. Bars represent means±SD. The different letter on the top of the column indicates a statistically significant difference between the treatments at P < 0.05 level using one-way ANOVA. Rice seedling subjected under the following six treatment vs C: Control; LN: Low doses of N-fertilizer; ON: High doses of N-fertilizer; CE: Control + *P. liquidambari*; LNE: Low doses of N-fertilizer + *P. liquidambari*; ONE: High doses of N-fertilizer + *P. liquidambari*.

**Figure 6:**
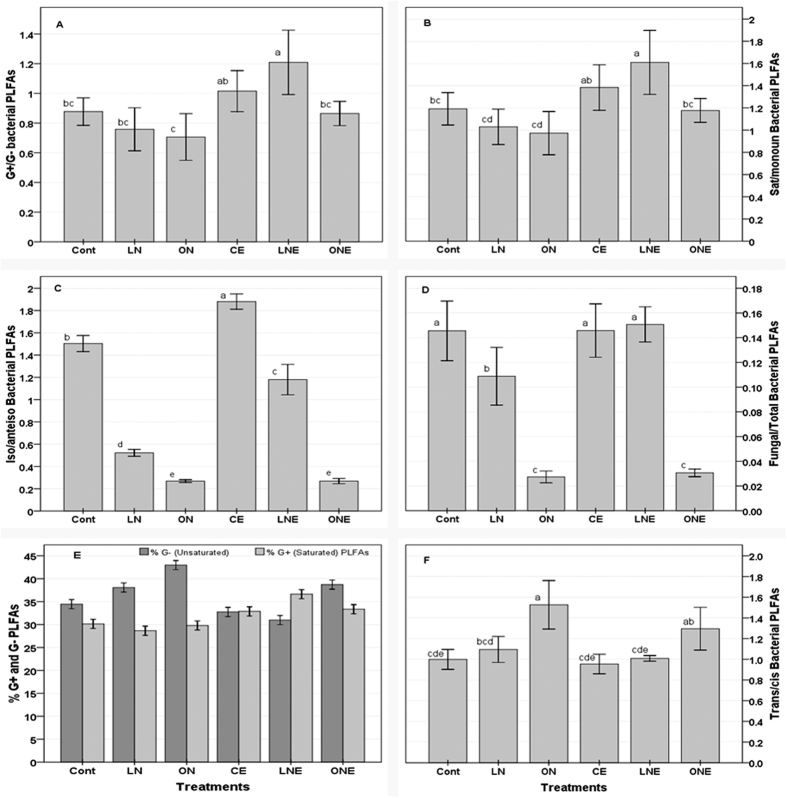
Comparison of microbial community structure (mean±SD, n = 3) of the rhizospheric soils of rice under different doses of N-fertilizer treatment with and/or without application of endophytic fungus *Phomopsis liquidambari* B3. Rice seedling subjected under the following six treatment vs C: Control; ON: Optimum N-fertilizer; LN: Low doses of N-fertilizer; CE: Control + *P. liquidambari*; LNE: Low N-fertilizer + *P. liquidambari*; ONE: Optimum N-fertilizer + *P. liquidambari*. G+/G, the ratios of gram-positive to gram-negative bacteria; Sat/mono, the ratios of normal saturated to monounsaturated fatty acids; Iso/anteiso, the ratios of iso- to anteiso-branched fatty acids; F/B, the ratios of fungi to bacteria; trans/cis, the ratios of trans to cis fatty acids. The different letter on the top of the column indicates a statistically significant difference between the treatments at P < 0.05 level using one-way ANOVA.

**Figure 7:**
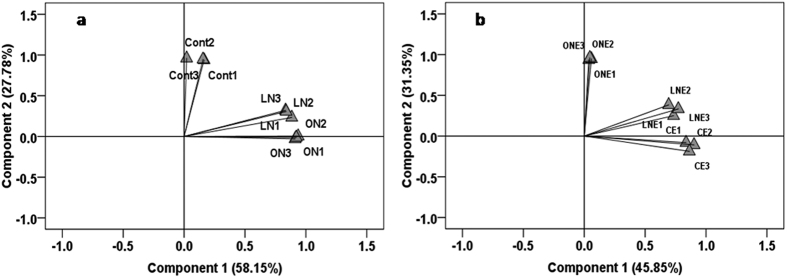
Discrimination of microbial community by principal component analyses (PCA) based on PLFA profiles of rice rhizosphere treated with different level of N-fertilizer and/or endophytic fungus *Phomopsis liquidambari*. Rice seedling subjected under the following six treatment vs C: Control; LN: Low doses of N-fertilizer; ON: High doses of N-fertilizer; CE: Control + *P. liquidambari*; LNE: Low doses of N-fertilizer + *P. liquidambari*; ONE: High doses of N-fertilizer + *P. liquidambari*.

The relative abundance of marker PLFAs specific to GB, G^−^ and G^+^ bacteria, actinomycetes and fungi and the ratios of G^+^ to G^−^ bacteria, sat to monouns, iso to anteiso and F to B represent the picture of application effect of N-fertilizer and *P. liquidambari* on microbial community structure and soil health condition (Figs 5 and 6). In general, the abundance of all the marker PLFAs belonging to different subgroup of microbe were higher in the rhizosphere of rice treated with low doses of N-fertilizer and *P. liquidambari* than the remaining treatment (Fig. 5A–F). Specifically, marker PLFAs of G^+^ bacteria as well as ratio of G^+^/G^−^, sat/mon and iso/anteiso was highest in LNE whereas highest G^−^ marker PLFAs and the ratio of G^+^/G^−^, sat/mon and iso/anteiso were lowest in ON soil compared to control (Figs 5C,D and 6A) which might be due to the application of high doses of N-fertilizer caused unbalanced the composition of soil nutrient pools or stress by lowering pH. Nutrient-rich conditions favor G^−^ bacteria^49^ and N-fertilization reduces soil pH and changes microbial community composition^7^,^8^. Dong *et al*.^29^ reported higher G^+^/G^–^ ratios in organic amendment than mineral fertilizer^29^. In general, G^−^ bacterial PLFAs was slightly higher than that of G^+^ bacteria in all the treatments irrespective of *P. liquidambari* inoculation (Fig. 5C,D). Similarly, Kimura and Asakawa^50^ found G^−^ bacteria dominate in the microbial communities of the floodwater and percolating water at Japanese rice fields^50^. The fungal marker PLFAs as well as F/B ratios decreased with increasing fertilizer doses which indicating the harmful effect of N-fertilizer on fungal community while *P. liquidambari* inoculation increased (Figs 5E and 6D). This might be due to the better soil environment by increasing C/N ratio through altered exudation of organic compounds by rice roots^3^. Mineral fertilizers reduce fungal PLFA and F/B ratio but organic manure with high C/N ratio stimulates fungi growth and increase F/B ratio^6^. Application of fertilizers decreased soil pH in planted rice under flooded condition^29^,^44^ therefore it can be concluded that N-fertilization decreased pH and reduced fungal growth. Highest F/B ratio was in LNE which suggesting sustainable agro-ecosystem with lower environmental impacts^51^. Similar to Dong *et al*.^41^ report; fertilizer applications enhanced actinomycetes populations^41^ (Fig. 5F) which might be due to the actinomycetes were less sensitive to changes in nutrient composition and environment. Different groups of microbes vary in their ability to adapt under various soil environment and fertilization certainly change the soil physical and chemical properties consequently directly or indirectly influence soil microbial community and activity^10^,^11^. However, trans/cis ratio increased with increasing level of N-fertilizer and highest was in ON whereas lowest in CE compared to control (Fig. 6F). *P. liquidambari* inoculation relatively reduced the trans/cis ratio in the N-fertilizer treated soil (Fig. 6F) which indicating the shift of trans to cis, stressed to non-stressed population. In general, G^+^/G^−^, sat/mon, iso/anteiso and F/B except tran/cis ratio decreased with increasing doses of N-fertilizer whereas *P. liquidambari* inoculation increased except tran/cis (Fig. 6). *P. liquidambari* addition to soil effectively alters the balance of soil microbes in the rhizosphere^3^,^26^. Microbial community alters as a result of fertilizer amendment, nutrient stress conditions^6^,^29^,^51^ and soil pH change^7^,^8^. PCA using identified PLFAs revealed a definite discrimination of microbial community belong to different doses of N-fertilizer and *P. liquidambari* inoculation (Fig. 7) and accounted for 85.93% (Fig. 7a) and 77.2% (Fig. 7b) of the total variance and generated three clusters. Control was mainly differentiated from LN and ON by the PC1; LN and ON were clearly separated by PC2 which explained 58.22% and 27.67% of the overall variance in the PLFA data, respectively (Fig. 7a). Though, PLFA patterns of LN were completely separated from the ON by the PC2 axis but somewhat close to each other which indicating that N-fertilizer treatments have more or less similar effect (Fig. 7a). In Fig. 7b, PLFAs of ONE were clearly different from both LNE and CE on PC1 axis with 45.85% variance and LNE from CE on the PC2 axis with 31.35% variance (Fig. 7b). Though, PLFA patterns of LNE are separated completely from the CE on the PC2 axis but somewhat close to each other than ONE which indicating that *P. liquidambari* inoculation reduced the influence of N-fertilizer on microbial community. PLFA profiles of the three replications were overlapped with each other, which suggested that rhizosphere of rice of each treatment had somewhat similar microbial community (Fig. 7a,b). Numerous reports showed distinct microbial community belongs to different treatment^6^,^29^,^51^. Hence, it is further evident that the rhizosphere microbial community structure of rice altered after application of different doses of N-fertilizer and *P. liquidambari*.

### Effect of different doses of N-fertilizer and plant growth promoting fungus *P. liquidambari* on nitrogenase activity

**Figure 8:**
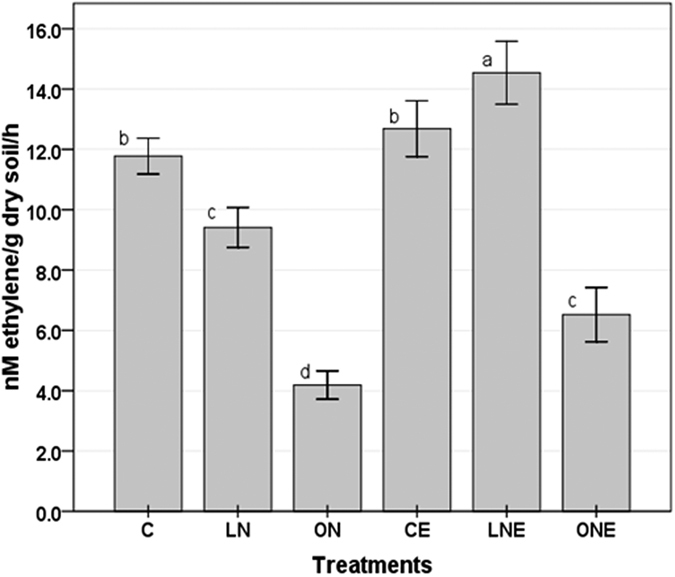
Acetylene reduction activity (ARA) of microbial community of rhizosphere soil of rice treated with different doses of N-fertilizer with or without inoculation of *Phomopsis liquidambari*. The values are means ± SD from three biological replicates. Different letters indicate statistically significant differences between treatments at P < 0.05. Rice seedling subjected under the following six treatment vs C: Control; LN: Low doses of N-fertilizer; ON: High doses of N-fertilizer; CE: Control + *P. liquidambari*; LNE: Low doses of N-fertilizer + *P. liquidambari*; ONE: High doses of N-fertilizer + *P. liquidambari*.

A considerable amount of N_2_ fixed by N-fixing microbes in many natural ecosystems and reduced fertilizer requirement. Rice benefited greatly from biological N-fixation in flooded rice fields^52^ and contributes 16–21% of rice N^53^. *In-vitro* nitrogenase activity assay revealed, low and high level of N-fertilization reduced nitrogenase activity of rice rhizosphere microbial community (Fig. 8) which might be due to the diazotorphic microbes used the available N for their rapid growth and metabolism rather than fixing N_2_ in soil and replaced by nondiazotrophs. Free-living N-fixing bacteria are typically facultative and N-fertilization suppressed N_2_ fixation^54^, abundance of N_2_-fixing bacteria^55^ and the reduced proportion of diazotrophs indicates competitive suppression by non-diazotrophs^56^. Nitrogenase activity reduced by around 20.09% and 64.43%, respectively in soil treated with low (1.25 g N per pot) and high (3.75 g N per pot) doses of N-fertilizer (Fig. 8). Application of more than 300 kg ha^−1^ N-fertilizer to a paddy soil completely inhibited N_2_ fixation^57^ and 100 to 150 kg ha^−1^ approximately 60%^58^. It is noticeable that the changes in the nitrogenase activity of N-fixing community caused by the fungal endophyte inoculation mainly occurred under low N-fertilizer conditions (Fig. 8). *P. liquidambari* colonization altered the exudation of organic compounds by rice roots^3^ which may increase C/N ratio. Increases C/N ratio favors conditions for N_2_ fixation^59^ and changes community structure and function. *P. liquidambari* inoculation at high doses of N-fertilizer increased nitrogenase activity of rhizosphere microbial community by 36% but remain very low compared to control (Fig. 8) which might be due to the inhibition of nitrogenase activity. Yang *et al*.^3^ also found relatively higher *nifH* gene copy numbers in high doses N-fertilizer and *P. liquidambari* treatment^3^. The capacity of N-fixation is unique in certain groups of microbes that contain highly conserved gene *nifH* which encodes nitrogenase enzyme^60^. Addition of NH_4_^+^ inhibits nitrogenase activity and probably followed the “NH_4_^+^ switched off” mechanism^61^. All diazotrophs live surrounding roots do not fix N_2_; some of them may be highly active but not abundant^62^. Therefore, mRNA-based studies are needed to identify active N-cycling bacterial species in our experimental conditions, and to further explore the effects of fungal endophyte on them.

### Effect of different doses of N-fertilizer and plant growth promoting fungus *P. liquidambari* inoculation on dry biomass, chlorophyll and nitrogen contents in rice

We conducted a pot experiments under field conditions and observed *P. liquidambari* and low doses of N-fertilizer or only high doses of N-fertilizer treated rice showed better growth, increased N, chla, chlb and root-shoot dry biomass content compared with remaining treatments (Supplementary Table S2). High doses of N-fertilizer treated plant should have high N-content but there were no significant differences with low doses N-fertilizer and *P. liquidambari* inoculation which may be due to the inoculation of *P. liquidambari* in rice facilitate better rhizosphere environment for N-fixation for N-fixing bacteria. Yang *et al*.^3^ found relatively higher *amoB* and *nifH* gene copy numbers in low doses N-fertilizer and *P. liquidambari* treatment^3^. In addition, *P. liquidambari* inoculation improve N-absorption, stimulates the expression of several genes involved in N-uptake and metabolism consequently enhanced N accumulation and N use efficiency^16^,^17^. Chlorophyll content is an indirect way of estimating productivity and understanding photosynthetic regime of plants. Similar to our results, chlorophyll contents increased in leaves by nitrogen supply^63^ and *P. liquidambari* inoculation^64^. Chla and chlb content of leaf were higher in low doses of N-fertilizer and *P. liquidambari* treatment compared to other but no significant differences with high doses N-fertilizer (Supplementary Table S2). The ratio of a/b is an indicator of greenness which represent the functional pigment equipment^65^; a/b = 2.5 is considered good for C3 plant for effective photosynthesis^66^ and lower than this caused reduction of greenness and photosysnthesis. The ratio of a/b were over 2.5 in LNE, ONE and ON which indicates undisturbed photosynthesis and improved growth compared to C, CE and LN (Supplementary Table S2). However, root and shoot biomass of *P. liquidambari* inoculated plants treated with low doses of N-fertilizer was higher than that of the uninoculated plants and there was no significant difference with high doses of N-fertilizer treatment (Supplementary Table S2). It means that rice plant showed better efficiency after treating with *P. liquidambari* in combination with lower doses of N-fertilizer and highlighting the importance of an active fungal symbiont for plant nutrient uptake in lower N-input agriculture. A number of our previous study demonstrated that application of N-fertilizers with *P. liquidambari* significantly enhanced N, Chla, biomass contents and growth and yield in rice under field condition^3^,^16^,^17^,^64^.

## Conclusion

In conclusion, different doses of N-fertilizer and *P. liquidambari* inoculation in rice had significant impacts on community level physiological profile, extracellular enzyme activities and specific PLFA markers related to specific microbial subgroup and total microbial community structure despite short duration of experiment. Our research adds new evidence in regard that application of *P. liquidambari* and low doses of N-fertilizer in rice stimulates substrate utilization and activities of all extra-cellular enzymes which represent changed in functional diversity toward positive direction. Marker PLFAs of five kinds of microbe; general bacteria, gram-positive, gram-negative, actinomycetes and fungi varied in the rhizosphere of rice soil in response to different level of N-fertilizer and *P. liquidambari* which indicate the adaptation ability of different group of microbes were different. High N-fertilizer application negatively affects nitrogenase activity representing diazotrophic population whereas *P. liquidambari* inoculation with low doses of N-fertilizer increased nitrogenase activity. Therefore, *P. liquidambari* inoculation and N-fertilizers application shift microbial functional groups in the opposite direction. *P. liquidambari* inoculation in combination with low doses of N-fertilizer increased biomass and growth of rice as like optimum doses of N-fertilizer which reduced the requirement of N-fertilizer, input cost and reduced environmental pollution caused by excess use of N-fertilizer. There were no differences in N and chlorophyll-content and growth of rice seedlings between high doses of N-fertilizer and low doses of N-fertilizer treatment inoculated with *P. liquidambari* which clearly demonstrated the beneficial effect of *P. liquidambari*. In short, application of *P. liquidambari* with low doses of N-fertilizer for rice cultivation; improved rice growth but reduced N-fertilizer requirement by increasing extra-cellular enzymes activities involved in C, N and P cycling, structural and functional diversity of microbes, nitrogenase activity involved in N_2_ fixation by free living diazotrophic microbes and accumulation of total N and chlorophyll content consequently photosynthesis”. The result of the present work along with our previous researches highlights the importance of the fungal endophyte for plant growth and nutrient uptake in lower N input crop production for sustainable agriculture.

## Experimental Procedures

### Study site and soil physicochemical analysis

Agricultural topsoil were collected from the surface layer (0–20 cm) of experimental rice fields of Nanjing Normal University (32° 6.318′N, 118° 54.88′E), Jiangsu Province, China. The region has a typical subtropical monsoon climate with mean annual precipitation of 1107 mm, temperature of 15.8 °C and relative humidity 83%. The soil for the experiment was yellow-brown loam; air-dried, mixed to homogeneity, sieved (2-mm mesh) to remove plant tissues, weighed and then added to plastic pots (25 cm diameter, 35 cm high). Soil moisture content was determined gravimetrically by weighing the soil sample, drying it in an oven at 105 °C for 24 h and then re-weighing the sample. The soil pH was 5.97, and the organic matter content was 1.08%. The nutrient composition of the soil was as follows: total N, 1.21 g kg^−1^: available N, 98.64 mg kg^−1^: total P, 0.39 g kg^−1^: available P, 24.67 mg kg^−1^: total K, 0.73 g kg^−1^: and available K, 67.58 mg kg^−1^.

### Fungal strain and rice seeds

The endophytic fungus *Phomopsis liquidambari* was isolated from the inner bark of *Bischofia polycarpa*^67^ an economically important woody tree plants belong to Euphorbeaceae family and native to southern China. The fungus was stored at 4 °C on potato dextrose agar (PDA, containing 200 g L^−1^ potato extract, 20 g L^−1^ glucose, and 20 g L^−1^ agar, pH 7.0). The rice cultivar used was a japonica subspecies of *Oryza sativa* L. “Wuyunjing 7”, which is a common cultivar grown in the Jiangsu Province of south-eastern China. The rice seeds were placed in 96% ethanol for 15 min, rinsed twice with sterile water, sterilized for 25 min in 0.1% HgCl_2_, and rinsed again six times in sterile distilled water^3^. The sterilized seeds and the last washing water were placed on PDA plates and cultivated for five days at 28 °C as sterility checks^3^,^16^,^17^.

### Fungal endophyte inoculation and cultivation of rice seedlings

*P. liquidambari* was cultured in 100 mL of potato dextrose broth in Erlenmeyer flasks (250 mL) for 3 days at 160 rpm in an orbital shaker at 28 °C, and then submerged fermentation was performed in 1-L Erlenmeyer flasks containing 500 mL of sterilized “PDB” and 10% seed culture broth for 4 days in the same condition. In total, 10 mL of culture broth was used to evaluate the dry cell weight by washing the mycelia with sterile distilled water twice and drying the mycelia in an oven at 80 °C to a constant dry weight. In total, 3.03 g (equivalent to 0.33 g dry weight) of fungal mycelia was collected, washed twice with sterile distilled water, and then diluted with sterilized water to a final volume of 200 mL. Since, our previous study by Yang *et al*.^3^ has confirmed colonization of *P. liquidambari* as endophyte in rice plant. Germinating rice seeds were treated by *P. liquidambari* suspension using as fungal agent to facilitate colonization^3^,^16^,^17^. The thoroughly sterilized seeds were randomly divided into two groups and transferred to Petri dishes (20 cm diam., 100 grains per dish). For the inoculated group (E+), 80 mL of the above-described fungal agent was added to each dish. The non-inoculated group (E−) was treated with 80 mL of sterilized deionized water as a control. Seeds were germinated and grown in Petri dishes for 4–5 days in a growth cabinet (30 °C during the day and 25 °C during the night, 16/8 h photoperiod at 250 mmol m^−2^ s^−1^). Germinated rice seeds were transplanted into pots (25 cm diameter, 35 cm high) containing 15 ± 0.5 kg of paddy soil suitable for raising seedlings. We first collected surface soil (0–20 cm depth) from agricultural field. Sun dried for about 7 days. Break the clod into small pieces removed debris and thoroughly mixed to make homogenous and reduced heterogenity. Then about 15 kg soils were put into the plastic pot and arranged those pot according to randomize complete block design (RCBD). For arranging pot, field was divided into three blocks and each block divided into 5 plots and each plot consisted of 15 pots. After 30 days of growth, seedlings at similar developmental stages were transplanted into pots (3 hills per pot and 1 seedling per hill and hill means a seedling with space provided for growing; hill to hill spacing was about 15 cm. Same soil were used in all pot) and grown in outdoor field under natural environment. As mentioned above that plants were arranged in RCB design, in which the main effects were the endophyte and the amount of N-fertilizer applied. N-fertilizer treatments consisted of two gradients: optimum doses of N i.e.; 3.75 g N per pot used as high doses of N (HN) and 1/3 of optimum doses i.e.; 1.25 g N per pot used as low doses of N (LN). Each treatment was replicated three times. N was applied as urea in two separate applications: the first application was made 7 days before planting (approximately 60% of total N), and successive applications were performed at the tillering stage (30% of total N). Phosphorus (1.6 g P_2_O_5_ per pot) and potassium (1.4 g K_2_O per pot) were applied as basal dressing before planting. Our previous study by Wang *et al*.^68^ found that endophytic fungus *P. liquidambari* survive in stress soil up to 30 days detected by real-time PCR^68^. We therefore *P. liquidambari* grown in “PDB” diluted by adding sterilized water (1:10) and approximately, 100 ml (15 g wet weight) were directly applied two time at the base of the growing rice seedling (previously inoculated) after transplanting to ensure endophytic colonization and survivability in the soil and to get maximum benefit from *P. liquidambari*. In addition, plants were regularly watered to maintain flooded condition during the whole growing season.

### Plant and soil sample collection and preparation

For the analysis of the functional and structural diversity of rhizosphere microbial community, soil samples were collected from each treatment at 9.00 am after 40 days of transplanting seedlings to the pots and placed in the field under natural condition. We first uprooted plant using shovel and excess soil were removed from surrounding area of root. Further, soil were removed as far as possible from root by hand because the soil was clay-loam type and tightly attached with root. Then the soils adhere with individual branches of root as well as the soils tightly attached on the root were collected as rhizosphere soil sample. Nine plants were sampled from each treatment. Each treatment had three replications. The soil samples of each replication were separately transported by plastic bag to the laboratory as quickly as possible. A portion of fresh soil was immediately used for community level physiological profiling described in the following section. The remaining rhizosphere soil samples were lyophilized (CHRIST LCG, Lyo chamber Guard, 121550 PMMA) till the soil moisture was in the range of 25–30%. Afterwards, the soil samples were homogenized, passed through a 2 mm sieve to clean and then maintained at −80 °C until enzyme activity and phospholipid extraction and PLFA assay.

### Community level physiological profiling (Biolog Ecoplate analysis)

Community level physiological profiles (CLPPs) were assessed by the Biolog EcoPlateTM system (Biolog Inc., CA, USA)^69^. Each 96-well BIOLOG Ecoplates consists of three replicates, each one comprising 31 sole carbon sources and water blank. Briefly, soil suspensions (soil 5 g, distilled water 45 ml) were shaken for 1 h and then pre-incubated for 18 h before inoculation to allow microbial utilization of any soluble organic compound from the soil^70^. Eight-fold dilution was performed and aliquots of 150 μl from a 10^−8^ diluted suspension were inoculated into the each well of the BIOLOG EcoPlates^71^. The plates were incubated at 25 °C for 5 days and color development was read as absorbance every 12 h with an automated plate reader (Biolog microstationTM, HAYWARD, CA94545, USA) at a wavelength of 590 and 750 nm.

### Soil extracellular enzyme activity assay

Activities of 8 soil enzymes were assayed as indicative of C-cycling: cellulase (EC 3.2.1.4) and β-glucosidase (Glu; EC 3.2.1.21)^72^,^73^; N-cycling: protease (EC 3.4.21-24), asparaginase and urease (EC 3.5.1.5)^74^,^75^; P-cycling: acid phosphatase (EC 3.1.3.2) and alkaline phosphomonoesterase (EC 3.1.3.1)^76^ and microbial activity: dehydrogenase (EC 1.1.1)^77^ using 1 g of lyophilized soil with their appropriate substrate and incubated for recommended duration and temperature at their natural pH. Enzyme activities were assayed in triplicate with one control, to which substrate was added after incubation and subtracted from the sample value.

### Nitrogenase activity of free-living N-fixing community (Acetylene Reduction Assay)

Nitrogenase activity was estimated using gas chromatography of ethylene formed by acetylene reduction activity (ARA) of diazotrophic microbial community of rhizosphere soil and expressed as acetylene reducing activity^78^. Commercially available standard ethylene was utilized to prepare standard curve for quantification, and vials with an equivalent volume of sterilized soil served as controls. By using an airtight syringe, 10% (v/v) of the air from the head phase of each bottle was removed and replaced with purified acetylene gas (99.8%). The cultures were incubated for 6 h for the reduction of acetylene (C_2_H_2_) to ethylene (C_2_H_4_) to take place. Gas samples (0.5 mL) were removed after 6 h and assayed for C_2_H_4_ production using a gas chromatograph (Agilent 6850, Gas Chromatograph) packed with a TM-PLOT-U column at 70 °C and equipped with a flame ionization detector. GC was adjusted at 40 °C, 120 °C and 220 °C with 50 °C min^−2^ for oven, injection and detection temperature, respectively. Carrier gas was He. Flow rate of He, H_2_ and Air were 1.1, 40 and 400 ml min^−1^. Make-up flow rate of N_2_ was 25 ml min^−1^. Under these conditions, retention time of ethylene was 1.54 min and the minimum concentration of ethylene detected was 0.1 ppm. The amount of ethylene was expressed as μmol of ethylene g^−1^ dry soil h^−1^.

### Phospholipid fatty acid (PLFA) analysis

Soil microbial community structure was characterized using PLFA analysis according to the modified Buyer method^6^. Five grams of lyophilized soil were placed in a 30 ml glass centrifuge tube with a Teflon-lined screw cap; 4 ml of 50 mM phosphate buffer (pH 7.4), 10 ml of methanol and 5 ml of chloroform were added. Tubes were sonicated for 10 m in a sonicating water bath at room temperature and then rotated end over end for 2 h at 28 °C. After centrifugation for 10 min at 700 × g, the liquid phase was transferred to a 30 ml test tube with Teflon-lined screw cap. For separation, another 5 ml of 50 mM phosphate buffer and 5 ml of chloroform were added then the mixture was shaken vigorously, and allowed to separate overnight in dark at room temperature. Aspirated top (aqueous) phase and the bottom (organic) phase was evaporated under N_2_ and stored at −20 °C. During drying the temperatures of water bath were maintained exactly at 30 °C. Lipid classes were separated by solid phase extraction (SPE) chromatography using a 500 mg silica gel column with 6 ml reservoir (Part No. 5982-2265, Agilent Technologies, Wilmington, DE, USA). Dried extract were dissolved in 250 μl chloroform and transferred to column then rinsed again 3 times using same amount chloroform and combined. Sample were let to drain into column and washed with 10 ml chloroform to removed/elute neutral lipids again washed twice with 5 ml acetone to remove/elute polar lipids. Phospholipids were eluted slowly with 5 ml methanol into screw-cap tube, evaporated under N_2_ at 30 °C and stored at −20 °C. Fatty acids were transesterified with 1 ml of 1:1 methanol:toluene and 1 ml of 0.2 M methanolic KOH and incubated for 15 min at 35 °C. After adding 2 ml of 1:4 chloroform:hexane, 1 ml of 1 M acetic acid and 2 ml of ddH_2_O, the mixture was vortexed and the phases allowed to separate for 5 min at 2000 rpm. The top phase was collected and the separation of bottom phase repeated with an additional 2 ml of 1:4 chloroform:hexane. Then, the combined organic phase was evaporated under N_2_ and stored at −20 °C. After that, samples were dissolved in 200 μl of hexane including 10 μg/ml methyl nonadecanoate (19:0) as standard and transferred to limited-volume GC vial before analysis. Since all fatty acids are temperature sensitive and some light and O_2_ sensitive. In general, extracts were kept in the dark as much as possible and did not heat above 30 °C during the evaporation stages. The fatty acid methyl esters were separated on a gas chromatograph with a flame ionization detector (Agilent 7890A Gas Chromatograph) using an HP-5 fused silica capillary column (30 m × 0.25 mm × 0.25 μm). Hydrogen gas was used as the carrier gas at 1.0 ml/min. At split ratio of 1:50, injection was employed. GC was adjusted at 40 °C, 230 °C and 270 °C for oven, injection and detection temperature, respectively and temperature program was 50 °C for 1 min, then 15 °C/min to 150 °C, maintained for 2 min, and then 3 °C/min to 250 °C with a final hold of 15 min. The individual PLFA peaks were identified according to the MIDI software using MIDI microbial calibration standards (Microbial ID, Inc., Newark, DE, USA). For each sample, different PLFAs were considered to be the representative of different groups of soil micro-organisms. All of the identified PLFAs were considered representative of the total PLFAs of the soil microbial community. The fatty acid nomenclature used is as follows: total number of carbon atoms:number of double bonds, followed by the position of the double bond from the methyl end of the molecule. *Cis* and *trans* geometry are indicated by the suffixes c and t. The branching is indicated as iso or anteiso, 10Me indicates a methyl group on the tenth carbon atom from the carboxyl end of the molecule, position of hydroxyl (OH) groups is noted, and cyclo indicates cyclopropane fatty acids. The lipid biomarker PLFAs used to represent the different microbial groups are as follows: C14:0, C15:0, C16:0, C17:0 and C18:0 for general bacterial; i14:0, i15:0, a15:0, a16:0, i17:0, a17:0, a18:0 for G^+^ bacteria; 16:1 2OH, 16:1w7c, 16:1w9c, 16:1w9t, 17:1w8c, Cyclo 17:0, 18:1w5c, 18:1w7c, 18:1w9t, and Cyclo 19:0 for G^−^ bacteria; 10Me16:0, 10Me17:0 and 10Me18:0 for actenomycetes and 16:1w5c, 18:1w9c, 18:3 w6c and 18:2 w9c for fungi^30^. All the PLFA biomarkers mentioned above were considered representative of total PLFA of soil microbial community. Ratio of F to B, G^−^ to G^+^, sat to unsat, trans to cis were used as indicator of shifts of microbial community structure^30^.

### Plant growth parameter

Plant sample were collected at the same time of soil sample collection. Rice roots and shoots of 10 plants were separated and washed, then placed in an oven at 105 °C for half an hour to inactivate the enzymes, and finally dried at 70 °C to a constant weight and recorded the dry weight. Shoot parts of the samples were separated, dried and analyzed for total N by the Kjeldahl N-determination method^79^. The remaining plant samples were immediately frozen in liquid nitrogen and separately stored at −80 °C until required. The content of chlorophyll *a* and *b* was determined according to Lichtenthaler and Buschmann^66^. Absorbance of the supernatant was recorded at 663.6 and 646.6 nm in spectraMax M2 and Chl*a* and Chl*b* levels calculated using the extinction coefficient.

### Statistical analysis

Statistical analyses were performed using SPSS 13.0 (SPSS, Chicago, IL, USA). The data on carbon substrate utilization, soil extracellular enzyme activity and PLFA content were subjected to the analysis of variance and pearson correlation. Least significant test (LSD) was calculated at the 0.05 probability level for making treatment mean comparisons. Box plots were graphed using the default settings of SPSS. Principal component analysis (PCA) were performed on the data sets of carbon substrate utilization and content of each individual maker PLFAs and canonical discriminant function analysis for extra-cellular enzymes activity to differentiate the effects of short-term fertilization and *P. liquidambari* inoculation on the microbial community structure and function of rhizosphere soil of rice using the SPSS software. Graphs were generated through Origin software version 8.0.

## Additional Information

**How to cite this article**: Siddikee, M. A. *et al*. Endophytic fungus *Phomopsis liquidambari* and different doses of N-fertilizer alter microbial community structure and function in rhizosphere of rice. *Sci. Rep.* **6**, 32270; doi: 10.1038/srep32270 (2016).

## Supplementary Material

Supplementary Information
