# Supplementary material for: Endophytic fungus Phomopsis liquidambari and different doses of N-fertilizer alter microbial community structure and function in rhizosphere of rice
Source: Sci Rep. 2016 Sep 6;6:32270. doi: 10.1038/srep32270 (PMC5011652; doi:10.1038/srep32270)
Supplement: Supplementary Information [file srep32270-s1.pdf]

Endophytic fungus *Phomopsis liquidambari* and different doses of N-fertilizer alter microbial community structure and function in rhizosphere of rice

Md Ashaduzzaman Siddikee, Mst Israt Zereen, Cai-feng Li and Chuan-Chao Dai*

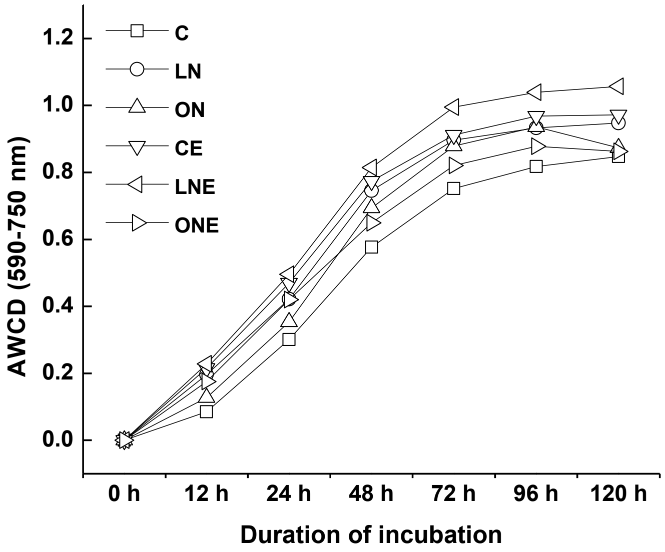

Supplementary Figure S1. Average well color development (AWCD) profiles (590-750 nm) for the microbial communities of rhizosphere soil of rice after short term treatment with different doses of N_2 fertilizer with and/or without inoculation of endophytic fungus *Phomopsis liquidambari* as evaluated in the Biolog EcoPlate incubated for 120 h. Rice seedling subjected under the following six treatment vs C: Control; LN: Low doses of N-fertilizer; ON: Optimum doses of N-fertilizer; CE: Control+ *P. liquidambari*; LNE: Low doses of N-fertilizer+*P. liquidambari*; ONE: Optimum doses of N-fertilizer+*P. liquidambari*.

Supplementary Table S1. Effect of different doses of N-fertilizer and endophytic fungus *Phomopsis liquidambari* inoculation on catabolic diversity of rhizosphere microbial community of rice as evaluated by average well-color development (AWCD), Shannon–Weaver diversity index (H) and substrate richness (S) in the Biolog EcoPlate incubated for 96 h.

Treatment	AWCD	H	S
C	0.29±0.019d	3.11±0.028b	25.33±2.906a
LN	0.44±0.016c	3.10±0.041b	24.44±1.018a
ON	0.40±0.016c	2.90±0.027c	18.11±0.694b
CE	0.41±0.013c	3.18±0.025a	25.56±0.192a
LNE	0.69±0.013a	3.22±0.027a	25.56±0.694a
ONE	0.51±0.022b	2.95±0.021c	19.00±0.577b

The value are means ± SD, N=3 for each treatment. Different letters indicate a significant difference at p≤0.0.5 level. Rice seedling subjected under the following six treatment vs C: Control; LN: Low doses of N-fertilizer; ON: Optimum doses of N-fertilizer; CE: Control+ *P. liquidambari*; LNE: Low doses of N-fertilizer+*P. liquidambari*; ONE: Optimum doses of N-fertilizer+*P. liquidambari*.

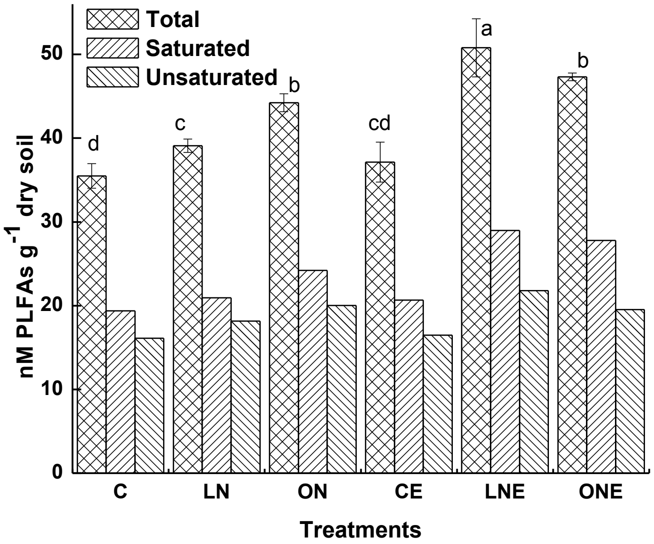

Supplementary Figure S2. Abundance of total PLFAs, saturated (GB, G^+ and actinomycetes) and unsaturated (G^- bacteria and fungi) PLFAs from soil of rice rhizosphere mapped as accumulated fractions on dependence of different doses of N-fertilizer application with or without *P. liquidambari* inoculation. Bars represent means±SD. The different letter on the top of the column indicates a statistically significant difference between the treatments at $P<0.05$ level using one-way ANOVA. Rice seedling subjected under the following six treatment vs C: Control; LN: Low doses of N-fertilizer; ON: Optimum doses of N-fertilizer; CE: Control+ *P. liquidambari*; LNE: Low doses of N-fertilizer+*P. liquidambari*, ONE: Optimum doses of N-fertilizer+*P. liquidambari*.

Supplementary Table S2. Effect of *Phomopsis liquidambari* inoculation on chlorophyll content, N-contents and growth of rice at tillering stages.

	RDW (g $plant^{-1}$)	SDW (g $plant^{-1}$)	N content (mg g^{-1})	Chlorophyll content (mg g^{-1} FW)		Chla/Chlb
				Chla	Chlb	
**Cont**	0.42±0.05d	2.07±0.23d	12.51± 0.85d	1.66±0.12d	0.78±0.07b	2.12
**LN**	1.19±0.03b	6.01±0.36b	28.54 ± 0.64b	2.49±0.06b	1.02±0.03a	2.44
**ON**	1.69±0.03a	8.46±0.47a	32.26 ± 0.84a	2.69±0.16a	1.02±0.12a	2.63
**CE**	0.68±0.02c	3.63±0.31c	16.51± 0.64c	1.88±0.07c	0.85±0.09b	2.19
**LNE**	1.68±0.01a	8.60±0.48a	32.08 ± 0.61a	2.80±0.11a	1.04±0.08a	2.68
**ONE**	1.72±0.07a	8.65±0.36a	33.19 ± 0.85a	2.79±0.10a	1.07±0.01a	2.64

The value are means ± SD, N=3 for each treatment. Different letters indicate a significant difference at p≤0.0.5 level. Rice seedling subjected under the following six treatment vs C: Control; LN: Low doses of N-fertilizer; ON: Optimum doses of N-fertilizer; CE: Control+ *P. liquidambari*; LNE: Low doses of N-fertilizer+ *P. liquidambari*; ONE: Optimum doses of N-fertilizer+*P. liquidambari*.
